# Alcohol Interaction with Cocaine, Methamphetamine, Opioids, Nicotine, Cannabis, and γ-Hydroxybutyric Acid

**DOI:** 10.3390/biomedicines7010016

**Published:** 2019-03-07

**Authors:** Ashok K. Singh

**Affiliations:** Department of Veterinary Population Medicine, College of Veterinary Medicine, University of Minnesota, St. Paul, MN 55108, USA; singh001@umn.edu; Tel.: +1-763-234-9655

**Keywords:** alcohol, addiction, withdrawal, cocaine, methamphetamine (METH), nicotine, marijuana, opioids, γ-aminobutyric acid (GABA)

## Abstract

Millions of people around the world drink alcoholic beverages to cope with the stress of modern lifestyle. Although moderate alcohol drinking may have some relaxing and euphoric effects, uncontrolled drinking exacerbates the problems associated with alcohol abuse that are exploding in quantity and intensity in the United States and around the world. Recently, mixing of alcohol with other drugs of abuse (such as opioids, cocaine, methamphetamine, nicotine, cannabis, and γ-hydroxybutyric acid) and medications has become an emerging trend, exacerbating the public health concerns. Mixing of alcohol with other drugs may additively or synergistically augment the seriousness of the adverse effects such as the withdrawal symptoms, cardiovascular disorders, liver damage, reproductive abnormalities, and behavioral abnormalities. Despite the seriousness of the situation, possible mechanisms underlying the interactions is not yet understood. This has been one of the key hindrances in developing effective treatments. Therefore, the aim of this article is to review the consequences of alcohol’s interaction with other drugs and decipher the underlying mechanisms.

## 1. Introduction

Ethanol (referred as alcohol hereafter) and other illicit drugs-of-abuse (referred as drug(s) hereafter) such as cocaine, methamphetamine (METH), nicotine, opioids, cannabis, and γ-hydroxybutyric acid (GHBA) continue to be a major public health concern globally. In 2015, the estimated global prevalence among the adult population was 18.4% for daily heavy alcohol use, 15.2% for daily tobacco smoking, 3.8% for cannabis, 0.77% for amphetamine/methamphetamine (METH) use, 0.37% for opioid use, and 0.35% for cocaine use [1]. Europe had the highest prevalence of heavy episodic alcohol use and daily tobacco use. Approximately 6.6% (16 million) of Americans aged 12 or older reported heavy drinking, 22.7% (55 million) reported binge drinking, and 8.1% (19.7 million) reported using drugs within the month prior to the survey [2]. However, drug abusers have historically tended to use more than one drug, a condition known as poly-drug abuse (defined as the concurrent or sequential abuse of more than one drug or type of drug, with dependence upon at least one [3]). Over the past several years, there has been an increasing tendency to combine narcotics, alcohol, sedatives, and/or stimulants [4,5].

Higgins et al. [6] have suggested that a combination of alcohol and other drugs of abuse such as cocaine, nicotine, opioids, or cannabis is popular among drug users, perhaps because of more intense feelings of ‘high’ beyond that perceived with either drug alone or less intense feelings of alcohol’s aversive effects. Their survey of the cocaine-dependent patients showed that more than half of the subjects met criteria for current alcohol dependence, and in more than 50% of the occasions both drugs had been used simultaneously. In forensic studies of Budd et al. [7] and Marzuk et al. [8], cocaine and ethanol were frequently identified in biological samples from fatally injured drivers. Dani and Harris [9] showed that almost 20 million cigarette-smoking Americans, either abuse or were addicted to alcohol. According to Patrick et al. [10], 94% of adults between the ages of 18 and 30 years have used alcohol in their lifetimes, and 56% have also used marijuana. In general, alcohol is commonly co-abused with (i) psycho-stimulants such as METH, cocaine or nicotine, (ii) opioids such as morphine, fentanyl and heroin, (iii) cannabis that is now legal in many states of the United States, and (iv) a potent neuro-inhibitor γ-hydroxybutyric acid (GHBA) [11,12,13]. The adverse effects of mixing alcohol with other drugs can be dramatically severe [14,15] that may hinder decision making, thinking, and neurocognitive capabilities [16,17,18,19,20].

**Figure 1:**
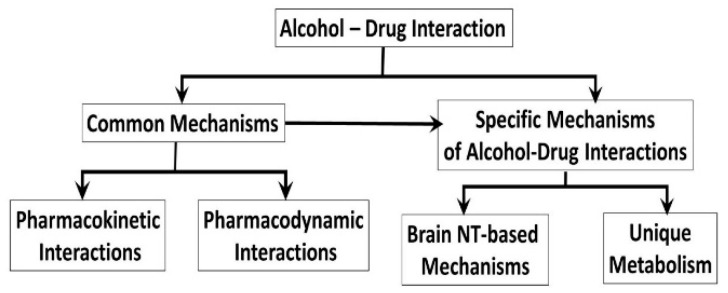
Proposed mechanisms for alcohol-drug interaction. NT: neurotransmitter systems and drug: cocaine, METH, nicotine, cannabis, opioids or GHBA.

Although mechanisms underlying the alcohol-drug interaction are not fully understood, two possibilities have been proposed: (i) common mechanisms including pharmacokinetics and pharmacodynamics [21,22] (Section 2) and (ii) specific mechanisms related to individual drug (Section 3). Earlier studies [23] have shown that alcohol increased the risk of heroin-related deaths, not due to any pharmacokinetic interaction, but due to pharmacodynamic interactions [22,23]. Conversely, alcohol modulated the effects of anti-inflammatory drugs via pharmacokinetic interactions [24]. Taken together, these observations indicate that an understanding of the alcohol-drug interaction may be essential to develop new strategies for treatment of addiction. Therefore, the aim of this review article is to decipher the common and drug-specific mechanisms underlying interaction between alcohol and cocaine, METH, nicotine, opioids, cannabis or GHBA. The overall hypothesis is that alcohol modulates the effects of cocaine, METH, nicotine, opioids, cannabis or GHBA via a common mechanism involving pharmacokinetics and pharmacodynamics, and/or drug-specific mechanisms addressed in Section 2 and Section 3, respectively (Figure 1).

## 2. Common Mechanisms of the Alcohol-Drug Interactions

People abusing alcohol or suffering from alcoholism tend to use multiple illegal and addictive drugs either sequentially or simultaneously [4,5]. Alcohol interacts with the co-abused drug and, additively or synergistically, modulate their effects via common pharmacokinetic (interference with the drug’s metabolism) and pharmacodynamic (modulation of the drug mechanisms) mechanisms detailed in the following sub-sections.

### 2.1. Pharmacokinetic Mechanisms of Alcohol-Drug Interactions

**Figure 2:**
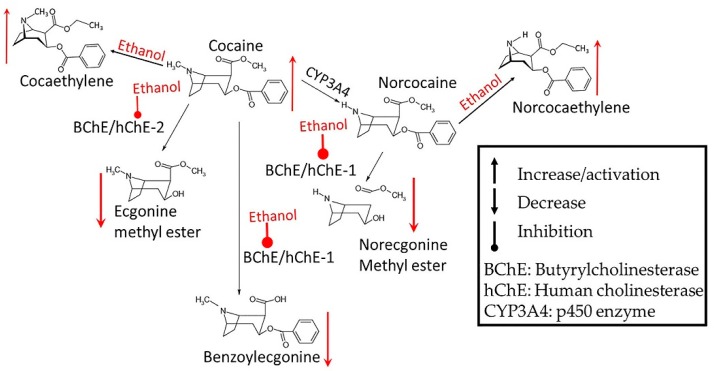
Effects of alcohol exposure on cocaine metabolism.

Alcohol, when ingested orally, undergoes substantial first-pass metabolism pre-systemically in stomach and systemically in liver [25]. Alcohol dehydrogenases (ADHs) and aldehyde dehydrogenases (ALDHs) metabolize alcohol into acetaldehyde and acetate, respectively [25]. In liver, alcohol induces the cytochrome P450 enzymes CYP2E1, CYP1A2 and CYP3A4 (ADH >> CYP2E1 = (CYP1A2 + CYP3A4)) that metabolize alcohol [26], many drug and pharmaceuticals [27,28]. Alcohol-induced modulation of these enzymes may also affect the drug’s pharmacokinetics (Figure 2, [11]). Accumulation of acetaldehyde may induce disulfiram-like reaction. In general, the following alcohol-drug interactions have been reported (Figure 2):

i. In the absence of alcohol, drugs are metabolized via liver CYP enzymes and the metabolites are excreted [11,29].
ii. Acute low dose of alcohol exposure in alcohol-naïve subjects is metabolized to acetaldehyde mostly by ADHs, but acute high-dose or chronic alcohol exposure may be metabolized by both ADH and CYP enzymes listed above. CYP enzymes remain induced in alcohol abstinent subjected chronically exposed to alcohol [11].
iii. In alcohol-naïve subjects using alcohol and another drug, acute dose of alcohol may compete with the drug for the same set of CYP enzymes and inhibit a drug’s metabolism. This may enhance the drug’s availability and ensuing increase in the harmful side effects from the drug [29].
iv. In recently abstinent chronic alcohol drinker, many drug-metabolizing CYPs remain induced, thus decreasing the drug’s availability and diminishing its effects for several weeks after drinking ceased. This suggests that a recently abstinent chronic drinker may need higher doses of medications than those required by nondrinkers to achieve therapeutic levels of certain drugs [30].
v. CYP enzymes activated by chronic alcohol consumption transform some drugs into toxic metabolites that can damage the liver or other organs [11].

These observations suggest that the drug and alcohol pharmacokinetics may play an important role on determining consequences of the alcohol-drug interaction. Earlier studies [30,31] have shown that the interaction pharmacokinetics can be predicted based on the metabolic profile of the drug. In general, alcohol exposure may modulate drug accumulation (C_max_ and AUC) by modulating their metabolism and excretion.

Parker and Laizurs [32] studied effects of alcohol on pharmacokinetics of cocaine administered via oral and intravenous (i.v.) administration (Table 1). They showed cocaine area under the curve (AUC_0–∞_) and benzoylecgonine (BE) AUC_0–∞_ values were approximately 5.5-fold and 2-fold, respectively, higher after i.v. compared with oral administration. Alcohol exposure significantly increased (3 to 4 folds) oral cocaine systemic bioavailability and peak concentration (C_max_) values, respectively, but alcohol did not affect oral cocaine elimination half-life. The BE AUC_0–∞_ values were approximately 2.5-fold higher with alcohol cocaine co-administration than with oral cocaine given alone. The mean cocaethylene concentration was 30.9 ± 7.3 ng/mL. Compared with oral cocaine administered alone, alcohol co-administration also reduced the AUC ratio by 40%. Alcohol did not significantly affect the AUC ratios for intravenous cocaine. Similar to the observations of Parker and Laizurs [32], Pan and Hedaya [33] also showed that alcohol exposure increased systemic bioavailability of intraperitoneal administered cocaine.

The pharmacokinetics of alcohol-cocaine interaction is determined by cocaine’s complex metabolic pathways (Figure 2) involving (i) pre first-pass and first-pass metabolism of cocaine to form BE and ecogonine methyl ester, (ii) conversion of cocaine to norcocaine by hepatic butyrylcholinesterase and p450 enzymes, and (iii) alcohol mediated formation of cocaethylene and norcocaethylene [34]. Patrick et al. [35] have shown alcohol to be a potent inhibitor of carboxyesterases and butyrylcholinesterase, resulting in accumulation of cocaine in the body. Parker et al. [32] have shown that alcohol suppressed first pass metabolism and elimination of cocaine. Taken together, these observations suggest that alcohol exposure may increase cocaine bioavailability and toxicity.

Alcohol, in addition to interacting with cocaine, also interacts with other drugs, albeit to different degrees. Li et al. [36] have shown that alcohol increased absorption and C_max_ of METH and its metabolite, amphetamine (AP) without altering their elimination. They also suggested that an alcohol-induced increase in toxicity of METH may be due to pharmacodynamics mechanisms. Adir et al. [37], Rose et al. [38] and Ferguson et al. [39] have provided indirect evidence that alcohol alters distribution and metabolism of nicotine, thus altering its toxicity. Cannabis and opioids, on the other hand poorly respond to alcohol exposure. Toenne et al. [40,41] have shown that alcohol increased half-life and decreased blood concentrations of cannabis but did not affect concentrations of its metabolites such as 11-OH- tetrahydrocannabinol (THC) and 11-nor-9-carboxy THC. Hartman et al. [42] and Lukas et al. [43] reported significant increases in THC and cannabidiol (CBD) concentrations, while two studies found no change. Likely, alcohol did not modify metabolism and pharmacokinetics of opioids.

### 2.2. Pharmacodynamics of Alcohol-Drug Interactions

**Figure 3:**
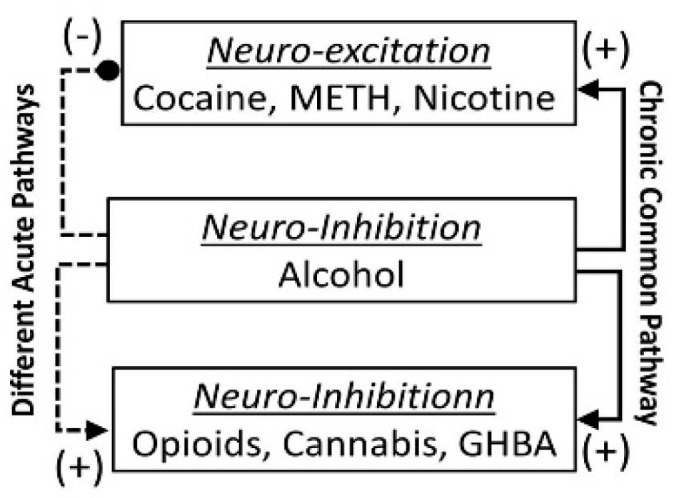
Proposed interaction between alcohol and Drug. (+): alcohol augments the effects, and (−): alcohol antagonizes the effects.

**Figure 4:**
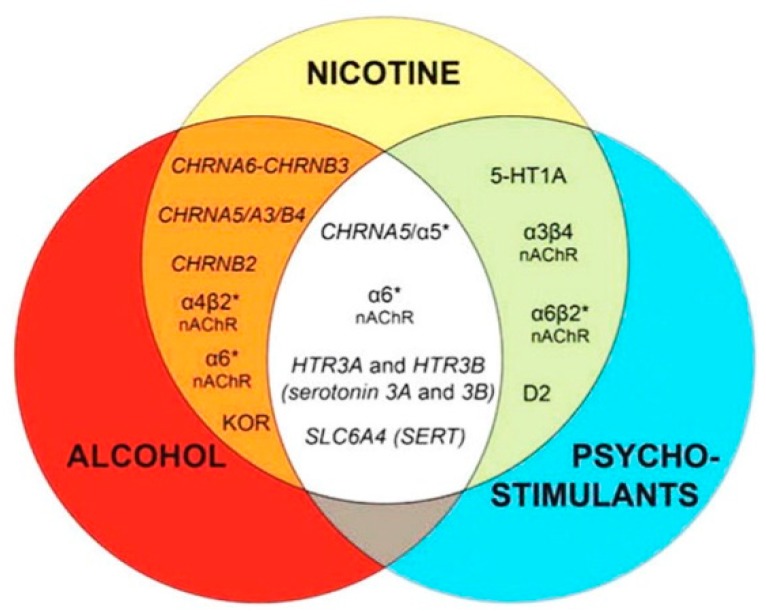
Overlapping receptor systems involved in nicotine and alcohol or psychostimulant dependence. Genetic and pharmacological studies in both humans and rodents suggest that co-use of nicotine and alcohol or psychostimulants is mediated, in part, by activity at overlapping substrates. In particular, cholinergic and serotonergic systems underlie reward-related behaviors, including drug intake, preference, and dependence to all three drugs of abuse. Common addiction genes are described by Cross et al [50] and Li and Burmeister [58]. Abbreviations: *: Nicotinic Acetylcholine Receptors (nAChRs) containing other subunits, ANKK1: ankyrin repeat and kinase domain 1, CHRN: cholinergic receptor nicotinic, C: CHRN, D2: dopamine receptors, Glu: glutamate, HTR: 5-hydroxytryptamine (serotonine) receptorts, KOR: kappa opioid receptor, NMDA: N-methyl-D-aspartate, SLC6A4: solute carrier family 6 member 4. Reproduced from [50] with permission.

Pharmacodynamics defines (i) the effects of alcohol and drug in body, especially at the target sites, and (ii) how drug combinations influence each other’s effects directly [44,45,46]. Figure 3 describes pharmacodynamic interactions of alcohol (a neuro-inhibitor) with neuro-stimulatory drugs (such as cocaine, METH or nicotine) and neuro-inhibitory drugs (such as opioid, cannabis and GHBA). In general, the following alcohol-drug pharmacodynamic interactions have been reported:

i. The acute *neuro-inhibitory* effects of the alcohol, opioids, cannabis and GHBA are caused via development of inhibitory postsynaptic potential (IPSP). The acute *neuro-excitatory* effects of cocaine, METH, and nicotine cause development of excitatory postsynaptic potential (EPSP) [11]. Therefore, acute alcohol exposure may attenuate the effects of neuro-stimulatory drugs but augments the effects of neuro-inhibitory drugs (Figure 4A). As an example, alcohol cause neuro-inhibition by inducing Cl^−^ influx into the neurons [47], resulting in development of neural membrane IPSP [48,49] that antagonizes the effects of stimulatory drugs, but additively or synergistically augment the effects of inhibitory drug.
ii. Chronic alcohol and drug exposure results in in development of tolerance and addiction via a common addiction mechanism (Figure 3). Therefore, chronic alcohol exposure may negatively impact addictive effects of both excitatory and inhibitory drugs.
iii. Figure 4 shows receptor overlap in development of alcohol, nicotine, and psycho-stimulant- (such as cocaine and METH) dependence. The genes listed in Figure 4 have received strong statistical and biological (knockout studies) support for association with multiple substances [50]. The nAChR gene variants such as gene cluster CHRNA5/A3/B4 encoding α3, β4, and α5 nAChR are associated strongly with poly-drug addiction [51,52,53,54]. The possible role of nAChR in alcohol dependence is further validated by the observation that varenicline, a partial agonist at α4β2 nAChRs and a full agonist at the α7 nAChR [55] reduced alcohol craving and total alcohol consumption in patients with alcohol use disorders [56,57].

A relatively older study conducted by Pan and Hedaya [33] have described negative effects of alcohol on pharmacodynamics of cocaine by using indices that relate brain cocaine concentrations with biological functions (Table 2). For example, E_max_ describes brain DA concentration measured at maximum change in the brain cocaine concentration (δC_max_), and EC_50_ describes a cocaine concentration that caused 50% E_max_ response. Similarly, the cardiac indices assessed rate constants for the direct effects of cocaine on cardiac functions. An increase in E_max_ indicates augmentation of DA release at δC_max_ cocaine concentrations. These studies showed that cocaine + saline administration increased the brain extracellular fluid (ECF) DA and nucleus accumbens (NAc) cocaine concentrations that peaked within 20–40 min, then gradually declined. Alcohol co-administration with cocaine caused significantly higher estimate for E_max_ values (not significant) but significantly lower IC_50_ values (Table 2). This suggests that alcohol exerts a direct stimulatory effect on the brain DA system in cocaine administered subjects. Unlike the neurological effects, the cardiovascular parameters were not different after cocaine + normal saline and cocaine + alcohol administration. This suggests that the same brain ECF cocaine concentration produced higher neurochemical response after co-administration of alcohol, causing more intense and longer lasting euphoric effects. This stronger response may be caused by the pharmacologically active metabolite cocaethylene [59].

Robinson et al. [60] have demonstrated that an antiepileptic drug, levetiracetam (LEV) that is a potent inhibitor of Glu-induced neuro-excitation, differentially modulated the effects of cocaine and alcohol. LEV pretreatment attenuated the development of locomotor sensitization to repeated alcohol exposure but enhanced both acute locomotor stimulation by cocaine and development of locomotor sensitization following repeated exposure. Although a possible mechanism underlying the ability of LEV to differentially modulate the effects of alcohol and cocaine is not fully understood, studies have proposed that the two substances may act at different sites of Glu_ergic_ neurotransmission in the mesocorticolimbic circuitry: alcohol and cocaine modulating Glu_ergic_ activities in VTA and NAc, respectively [61]. Acute cocaine administration stimulated Glu release in NAc, but not in VTA [62], while acute alcohol increased the firing rate of DA_ergic_ VTA neurons [63]. These differences may explain in part why LEV blocks the development of locomotor sensitization to alcohol but not to cocaine, despite observations that increased Glu_ergic_ sensitivity of DA_ergic_ VTA neurons is an early triggering event in sensitization to both cocaine and alcohol [64].

Taken together, these observations suggest that the neuro-inhibitory and neuro-excitatory substances may cause acute effects by diverse mechanisms, but chronic addictive effects via a common mechanism. Alcohol may augment the acute effects of neuro-inhibitory but attenuate the acute effects of neuro-excitatory drug. However, alcohol may augment the addictive effects of both groups of drugs.

## 3. Specific Alcohol-Drug Interactions

As discussed earlier, the brain neurotransmitter (NTs) systems including, but not limited to, endogenous opioids (eOPs), DA, GABA, glutamate (Glu), glycine (Gly), serotonin (5-HT), excitatory amino acids (EAAs) and their respective receptors play important roles in rewards, aversive effects and addictive effects of alcohol and other drugs [65]. In drug-free situations, all NTs interact with each other (positively (+) or negatively (-) as shown in Table 3) and maintain a NT balance. Depending on the type of substance (alcohol, cocaine, METH, nicotine, opioids, cannabis, and GHBA), different groups of NTs have been suggested as direct targets. For example, GABA and Glu are key targets of alcohol that simultaneously increases inhibitory neurotransmission through GABA and reduces excitatory neurotransmission through Glu [66]. Unlike alcohol, DA and ACh are direct targets for amphetamine and nicotine, respectively. Amphetamine directly increases the DA level in the synaptic cleft [67], whereas nicotine mimics psychopharmacological effects of ACh and modulates DA release [68,69]. In addition to the direct effects, addictive substances can also modulate other NTs through indirect pathways. For example, alcohol’s direct effect on the striatum Glu may modulate, GABA_ergic_ activity in the *NAc*. Direct and indirect mechanisms both may play an important role in alcohol-Drug interactions.

**Figure 5:**
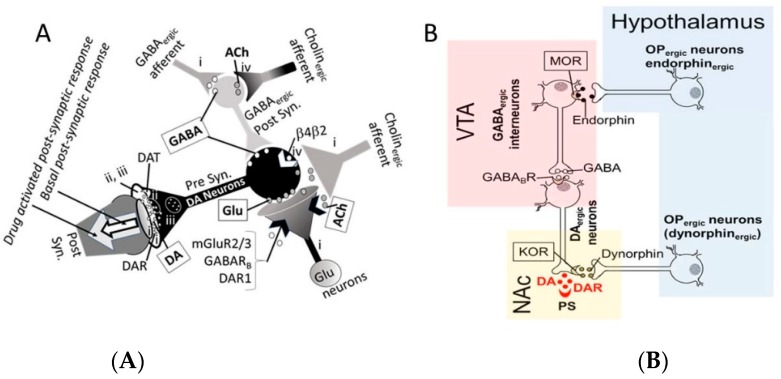
(**A**) Possible roles of Glu_ergic_, GABA_ergic_ and ACh_ergic_ neurons in regulation of ventral tegmental area (VTA) DA_ergic_ neuron excitability. Opioid (OP) receptors are not shown in this diagram). The presynaptic DA_ergic_ neurons (1) express GABA_B_R_,_ DAR1, mGluR2/3 and nicotinic (α7 and α4β2) receptors thus the neuronal activity is modulated by Glu, GABA, ACh and nicotine, and (2) received signals form excitatory Glu_ergic_ neurons releasing Glu, excitatory Cholin_ergic_ neurons releasing ACh, and inhibitory GABA_ergic_ neurons releasing GABA. The released NTs bind to their respective receptors on DA_ergic_ neurons and elicit excitatory (depolarization) or inhibitory (hyperpolarization) response. The Glu_ergic_ neurons also express mGluR2/3, AChR, GABA_B_R and DAR1 receptors, thus the neuronal activity is modulated by Glu, GABA and ACh. The GABA_ergic_ interneurons receive receptors signals from GABA_ergic_ and Cholin_ergic_ efferent. (**B**) Interaction of OP_ergic_ neurons (endorphin_ergic_ neurons release endorphin, a MOR agonist, while dynorphin_ergic_ neurons release dynorphin, a KOR agonist) with GABA_ergic_ interneurons and DA_ergic_ neurons. The DA_ergic_ neurons from the VTA project to ANc and are under tonic inhibition by GABA_ergic_ interneurons that are under direct inhibition by endorphin_ergic_ neurons from the hypothalamus Therefore, stimulation of endorphin release in VTA inhibits GABA_ergic_ interneurons and ensuing disinhibition of the DA_ergic_ neurons, leading to increased DA release in NAc. Acute alcohol stimulates endorphin and met-enkephalin release, leading to an increase in DA release, while chronic alcohol stimulates dynorphin release that could attenuate DA release in the NAc. Abbreviations: DA: dopamine, DAR: DA receptors, DAT: DA transporter, Glu: glutamate, and α7 and α4β2: nicotinic receptors. The VTA DA_ergic_ neurons receive signals from Glu_ergic_ (releases excitatory neurotransmitter, Glu), Cholin_ergic_ (releases excitatory NT, Ach), and GABA interneurons (releases inhibitory neurotransmitter, GABA) that receive signals from GABA_ergic_ (releases GABA) and Cholin_ergic_ (releases ACh) neurons. Binding of GABA to its receptors on. GABA_ergic_ afferent neurons releases GABA inhibits GABA interneurons, thus activating the DA_ergic_ neurons. Symbol-i: the sites of actions for alcohol, symbol-ii: the site of action for cocaine, and symbol-iii: the site of action for nicotine.

An interactive mechanistic diagram showing possible roles of the brain NT and receptor systems in different brain regions are shown in Figure 5A. The details are discussed in the figure legend. As shown in Figure 5B, a direct alcohol-induced activation of hypothalamus OP_ergic_ neurons may indirectly modulate GABA_ergic_ neurons followed by modulation of DA_ergic_ neurons. This may modulate the effects of amphetamine that acts by directly activating DA_ergic_ neurons. Thus, direct and indirect effects of alcohol may modulate effects of co-administered drug. The following paragraph includes a brief discussion of the overall mechanism of action of alcohol.

Acute alcohol exposure, in addition to activating the alcohol metabolizing enzymes such as alcohol dehydrogenase (ADH), acetaldehyde dehydrogenase (ALDH) and microsomal P450 enzymes, also causes a psychotropic depression of the CNS, leading to various behavioral and biological alterations [25,26]. Alcohol-induced depression is causally related to (i) direct increase in GABA_ergic_ activities, (ii) direct decrease in Glu_ergic_ activities and associated decrease the intracellular concentration of calcium ions (Ca^2+^), and (iii) indirect modulation of DA_ergic_, 5HT_ergic_ and ACh_ergic_ activities [71,72,73]. All processes, except the DA_ergic_ activity, may be negatively regulated by acute alcohol exposure [74]. In contrast, chronic alcohol use causes tolerance and addiction by (i) down-regulating GABA receptors and phosphorylation of ERK which is regulated by GABA receptors, and (ii) activating Glu receptors in the hippocampus that is involved in seizures development during alcohol withdrawal [74,75,76]. Chronic alcohol abuse may also prevent activation of the memory circuit and the explicit memory supported by the hippocampus [77]. Alcohol withdrawal in addicted subjects decreases GABAR but increases GluR activities, resulting in strong neuro-stimulation (red arrows). Figure 5B shows multiple neurotransmitters and neuromodulators that collectively mediate the reward-profile of alcohol [78]. In general, alcohol directly modulated OP_ergic_, GABA_ergic_ and Glu_ergic_, and indirectly modulated DA_ergic_, 5-HT_ergic_ and cholinergic (ACh_ergic_) presynaptic neurons, thus modulating the release of neurotransmitters and ensuing modulation of postsynaptic neurons. Binding of neurotransmitters to the postsynaptic receptors release may modulate respective behavioral traits.

These observations suggest that acute and chronic alcohol exposure may target different sets of the CNS NTs and, therefore, differently modulate the effects of excitatory and inhibitory drug. Acute alcohol exposure, due to its depressive effects, may augment the effects of neuro-inhibitory drugs (cannabis or GHBA), but suppress the effects of neuro-stimulatory drugs (cocaine, METH and nicotine). However, chronic alcohol exposure may augment the neuro-stimulatory drugs but suppressing neuro-inhibitory Drugs. Interaction of alcohol with other drugs are discussed below.

### 3.1. Alcohol-Cocaine Interaction

**Figure 6:**
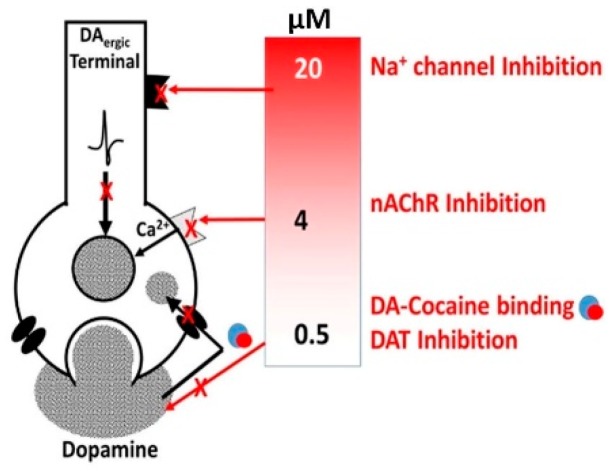
Diagram showing that cocaine may inhibit DAT-mediated DA uptake and DA release via nAChRs and Na^+^ channels in a dose-dependent manner.

Cocaine is a powerful addictive, psychoactive, stimulant drug illegally available on the streets as a fine, white powder. Whatever the form, cocaine acts as a strong stimulant substance that can (i) provide a rapid-onset of rewarding high, (ii) speed up various physiologic processes via its CNS effects, and (iii) influence both short- and long-term mental health. Acevedo-Rodriguez et al. [79] have shown that cocaine, at concentrations around 0.5 µM that is readily achievable in cocaine abusers, inhibited the DA transporter (DAT)-mediated uptake of DA. At concentration around 4 µM, cocaine inhibited nAChRs and altered DA release [80]. At cocaine level ≥20 μM, its anesthetic effect may be triggered (Figure 6). This suggests that the mechanistic effects shown in Figure 6 may contribute to the cocaine-induced increases the ratio of phasic to tonic DA release and thus potentially enhances its reinforcing abilities [81].

**Figure 7:**
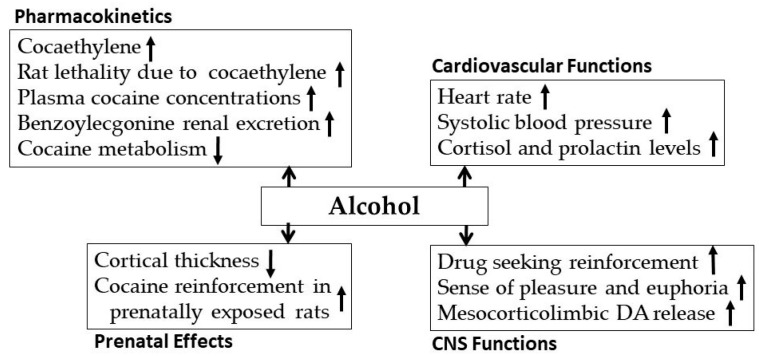
Effects of alcohol exposure on cocaine’s pharmacokinetics, cardiovascular function, CNS functions and prenatal effects.

Epidemiological studies have shown that, compared to the control subject (cocaine free), the prevalence of alcohol use was found 89% higher among cocaine dependents [82,83], possibly due to the perception of higher increase of reward effects when alcohol and cocaine were co-administered compared to either drug administered alone [84,85,86]. In a study conducted on rats, intravenous injections of cocaine increased alcohol drinking, suggesting that cocaine potentiated alcohol seeking [87]. A preclinical study has shown a higher susceptibility of the reinforcing effects of cocaine in selectively bred alcohol preferring (P) rats compared to its outbred Wister rats, suggesting a higher sensitivity of alcoholics to the reinforcing effects of cocaine [88] Similarly, it has been revealed that genetically predisposed subjects for alcohol dependence have a higher rate to be cocaine dependents [89]. This suggests that alcohol and cocaine, when co-administered, potentiate the effects of individual drugs. Different aspect of interaction between alcohol and cocaine exposure are shown in Figure 7 and described below.

Cocaine and alcohol co-administration generates a unique metabolite, cocaethylene that is equipotent in inhibition of binding to the dopamine and serotonin reuptake complex [90,91]. Cocaethylene may be less anxiogenic and more reinforcing [92,93], but it is more lethal than cocaine [94] Concurrent use of cocaine and alcohol has been associated with greater risk of sudden death than after cocaine alone [95]. Cocaethylene has been detected in wastewater, an observation that has been used as evidence of cocaine and alcohol co-abuse in urban area. In addition, cocaethylene concentrations in wastewater was significantly higher during weekends compared to weekdays, further suggesting a higher co-abuse of cocaine and alcohol [96].

Alcohol administration has been shown to increase the plasma concentration of cocaine [97], leading to an increase in cocaethylene concentration in plasma and decrease in benzoylecgonine renal excretion [98]. Although alcohol ingestion did not alter cocaine half-life, it significantly increased cocaethylene’s half-life [99], thus increasing the exposure to cocaethylene’s deteriorating toxic effects. Cocaine and alcohol co-exposure also has deleterious effects on cardiovascular and endocrine systems as evidenced by an increase in heart rate, systolic blood pressure, cortisol, and prolactin concentrations, and cerebral blood perfusion [100]. It has been shown that cerebral hypo-perfusion was more common among individuals taking cocaine and alcohol together compared to individuals taking cocaine or alcohol alone [101,102].

Several indices of neuropsychological performances such as intelligence, memory, verbal learning were found to be negatively affected by the concurrent intake of cocaine and alcohol compared to either drug administered alone [103,104]. The sense of pleasure and euphoria increased in co-abuse of alcohol and cocaine and consequently elevated the risk of dependence and toxicity [105]. Alcohol and cocaine co-exposure increased extracellular DA concentration in the NAc, a region involved in the rewarding and reinforcing effects of drugs of abuse [106,107,108], compared to either drug administered alone in rats [109]. One recent study has demonstrated a significant interaction in prenatal co-exposure of cocaine and alcohol on cortical thickness in youths prenatally exposed to these drugs [110,111].

### 3.2. Alcohol-Methamphetamine Interactions

**Figure 8:**
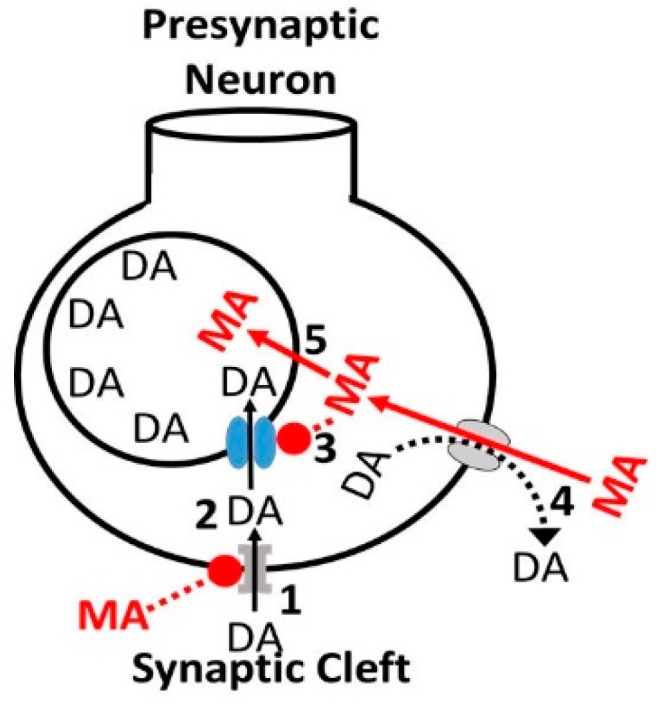
Mechanism of action of METH on DA neurotransmission. [113. 1: Methamphetamine (MA) inhibits DA reuptake, 2: MA phosphorylates DAT resulting in its internalization, 3: MA inhibits DA’s synaptosomal uptake via vesicular monoamine-*transporter* 2 (VMAT-2), 4: intracellular uptake of MA reverse transports DA via DAT into the synaptic cleft, 5: MA diffuses into the synaptosome impairing DA storage.

METH’s main mechanism of action is its ability to increase in neuronal release of DA into the NAc, an effect mediated via alterations in both the DAT and the vesicular monoamine transporter-2 (VMAT-2) [112]. In addition, METH phosphorylates DAT via protein kinase C leads to internalization of DAT, thus impairing the normal function of DAT [113]. Concurrent with reuptake inhibition, METH also induces DA efflux into the synapse (Figure 8).

**Figure 9:**
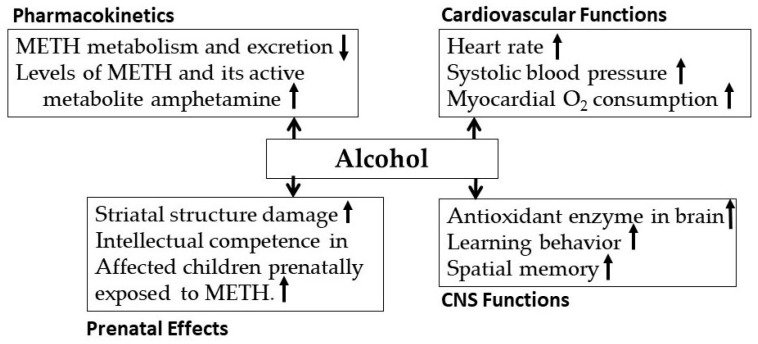
Effects of METH exposure on alcohol’s pharmacokinetics, cardiovascular function, CNS functions and prenatal effects.

Alcohol and METH, often used together, cause co-morbid disorder [113,114]. Approximately 77% of people diagnosed with amphetamine dependence also have an alcohol use disorder [115,116]. Within the population of METH users, alcohol consumption increases the probability of METH use by four-fold [117,118,119]. Figure 9 shows possible effects of concurrent alcohol and METH exposure. METH abusers frequently use alcohol to have a higher level of euphoric effects. But, alcohol may inhibit METH metabolism, resulting in higher blood METH concentration, with an increase in its stimulating effects on brain and heart, resulting in significant negative effects on mood, performance, and physiological behaviors [120]. Co-exposure to alcohol and METH also resulted in (i) synergistic depletions of DAT, SERT, and DA and 5HT content, and (ii) increase in LPS and COX-2 in rats [118,121]. This suggests that prior alcohol drinking may also increase the inflammatory mediators, thus enhancing neurotoxicity.

Mendelson et al. [122] in humans and Wells et al. [123] in mouse have shown that in utero exposure of a combination of alcohol and METH may cause greater toxicity in offspring than either alcohol or METH. This interaction may be due to the increased production of reactive oxygen species (ROS) that alter signal transduction, and/or oxidative stress-induced damage to cellular macromolecules like lipids, proteins, and DNA, the latter leading to altered gene expression [123]. This may be causally related to the development of cardiac cytotoxicity associated with adverse cardiovascular effects. Andez-Lopez et al. [124] have shown that, in addition to alcohol-METH combination, the 3,4-Methylenedioxy-methamphetamine (Ecstasy) and alcohol combination also augmented euphoria and wellbeing than Ecstasy or alcohol alone. Subjects may feel euphoric and less sedated and might have the feeling of doing better, but actual performance ability continues to be impaired by the effect of alcohol.

### 3.3. Nicotine

**Figure 10:**
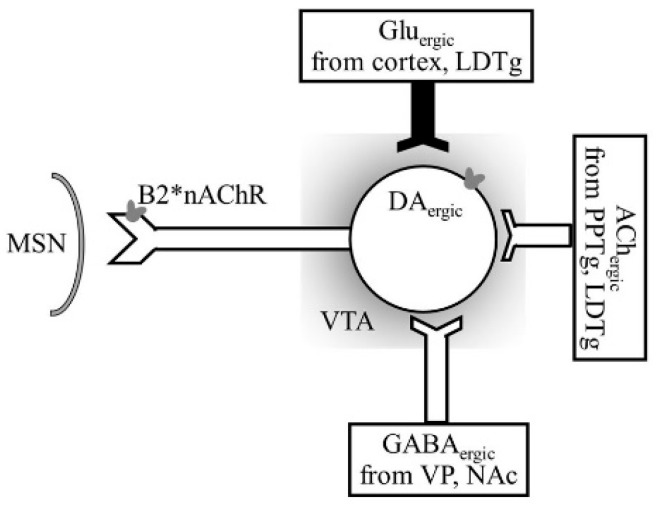
A schematic representation of the ACh_ergic_ signaling in VTA and afferents. The DA_ergic_ output neuron contains high-affinity b2-nAChRs that are inhibited by GABA releasing from GABA_ergic_ interneurons containing high-affinity b2-nAChRs. GABA_ergic_, Glu_ergic_, and ACh_ergic_ projections arrive from the ventral pallidum (VP), the laterodorsal tegmental nucleus and LDTg/PPTg pontine tegmental nuclei, respectively. DA_ergic_ neurons release DA in the NAc, which show biochemical alterations after alcohol exposure.

Nicotine is a highly addictive substance of tobacco, acting via binding to the nicotinic acetylcholine (ACh) receptors or nAChRs that respond to the neurotransmitter ACh. Nicotine addiction is mediated through nAChR expressed on most neurons in the brain. Tolu and Eddine [125] showed that nAChR-mediated activation of GABA neurons in the VTA plays a crucial role in the control of nicotine-elicited DA_ergic_ activity (Figure 10). DA and GABA make a concerted effort to generate reinforcing actions of nicotine through DA_ergic_ neurons. Therefore, GABA_ergic_ neurons may be a potential drug development target for cessation of drug development.

**Figure 11:**
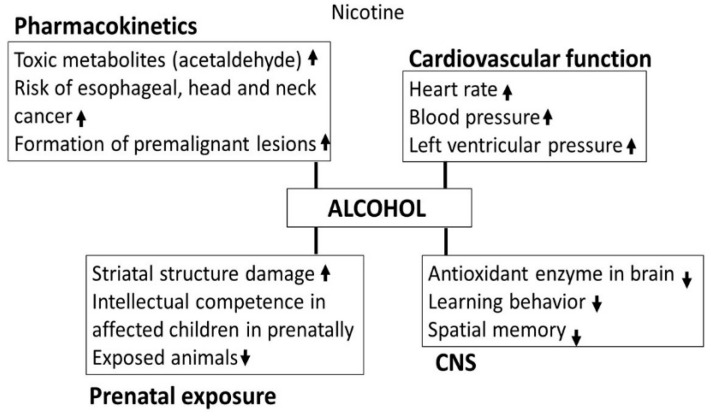
Effects of alcohol exposure on nicotine’s pharmacokinetics, cardiovascular function, CNS functions and prenatal effects.

Alcohol and cigarette smoking is the most common practice globally that may be most costly in terms of health and societal costs [126,127,128]. Nicotine dependents may have a high tendency to be alcohol dependents [129]. It has been reported that more than 80% of chronic alcohol users are also smokers [130,131,132]. In a preclinical study, rats chronically co-exposed to alcohol and nicotine showed higher nicotine self-administration as compared to drug self-administered alone [133]. The overall effects of alcohol-nicotine interaction are shown in Figure 11.

Blomqvist et al. [134,135] have proposed that alcohol modulates the reinforcing effects of nicotine by directly interacting with the nAChRs, β2 and β4 [136,137]. Lüscher and Malenka [138] have shown that chronic nicotine exposure triggers a conformational change in β4 nAChRs that initiates various forms of synaptic plasticity and modify the VTA-DA neuron’s responses to alcohol and alcohol drinking behaviors. Norbinaltorphamine (norBNI), a KOR antagonist, robustly increased alcohol and nicotine self-administration in adult male rats but not in female rats [139,140]. Taken together, these findings suggest that nicotine, from either tobacco or e-cigarette use, may increase the vulnerability of teenage boys to alcohol abuse.

### 3.4. Alcohol-Opioid Interactions

Opioids, addictive substances derived from the poppy seedpod, occurs as (i) a natural drug such as opium, morphine and codeine, and (ii) a synthetic drug such as dilaudid, demerol, oxycodone, vicodin, fentanyl, methadone or heroin. Opioids are commonly used analgesic agent with potential for abuse as street drug [141,142]. In body, the opioid drugs compete with the receptors for endogenous opioid peptides (eOP) such as β-endorphin, enkephalins, and dynorphins released by selective OP_ergic_ neurons. The eOPs, nOPs and sOPs bind to three families of opioid receptors (OPRs): μ (MOR), δ (DOR), and κ (KOR) with differing affinities [143]. The three OP receptors (OPRs) are widely distributed in the brain regions involved in pain modulation, reward, stress responses, and autonomic control [144]. OPRs selectively interact with G-proteins (composed of two subunits, Gα_i_, α_s_ or α_o_ and βγ subunits) and form MOR-Gα_i_βγ for β-endorphin and endomorphin 1, DOR-Gα_o_βγ for enkephalins, and KOR-Gα_s_βγ for dynorphin and endomorphin 2 [145]. The OPR-G protein complex (such as MOR-Gα_i_βγ), upon binding to an eOP or a sOP, dissociates into MOR-Gα_i_ subunit and βγ subunits. MOR-Gα_i_ subunit directly inhibits adenylyl cyclase (AC) that reduces cAMP formation and activates inwardly rectifying K^+^ channels (GIRK^+^), causing neuro-inhibition and ensuing analgesic response [146]. The βγ dimer directly inhibits voltage-dependent Ca^2+^ channels [147]. Taken together, these changes block the presynaptic signal from activating postsynaptic terminal, thus causing analgesia.

Acute alcohol exposure has been shown to potentiate the opioid-induced increase in analgesia and CNS depression, leading to serious side effects including respiratory distress, coma, and death [148,149]. Chronic alcohol exposure may develop coaddiction when addiction to one drug (such as an opioid) enhances craving for another such as alcohol [150,151,152,153,154,155]. A Canadian study has shown that approximately 82% of apparent opioid-related deaths from 2016 to 2017 also involved one or more type of non-opioid substances including alcohol [148]. Polettini et al. [156] have shown that heroin, a dangerous illegal opioid, can interact with alcohol and produce a sensation of greater pleasure than the two individually, while at the same time inhibiting the respiratory system. In addition, alcohol may exacerbate the neuronal situation by inhibiting heroin metabolism (pharmacokinetic mechanism) [157]. Despite the seriousness of the alcohol-opioid interaction, the underlying mechanisms are not fully understood. Therefore, the aim of proceeding sub-sections are to discuss combined effects of alcohol and opioid on analgesia, CNS inhibition and addiction.

**Figure 12:**
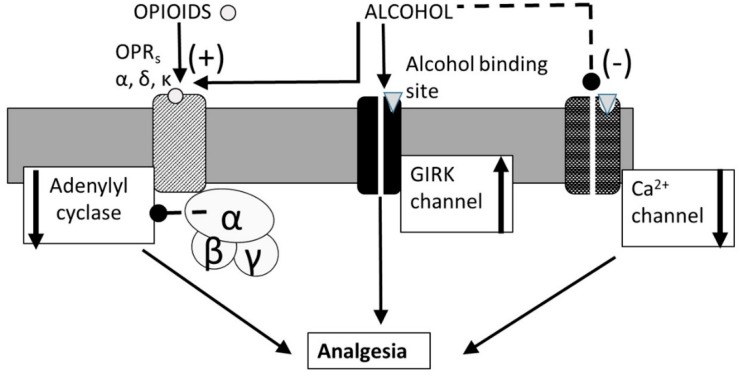
Signal pathways mediating opioid-induced analgesia. Opioids distinctively activates MOR, DOR, and KOR, that leads to G_i/o_ protein activation. The activated G_i/o_ protein activates the GIRK channel and inhibits the function of adenylyl cyclase and calcium channels. Alcohol activates the GIRK channel directly and modulates the functions of other target molecules. Non-steroid anti-inflammatory drugs (NSAIDs) induce analgesia in a GIRK channel independent fashion. In weaver mutant mice, GIRK channel activation either by G_i/o_ protein or by alcohol is impaired, and both opioid- and alcohol-induced analgesia is reduced, whereas NSAIDs normally induce analgesia.

Figure 12 shows signaling pathways for analgesic effects of opioids and effects of alcohol drinking on it. In addition to the OPRs, type-2 G-protein coupled inwardly rectifying potassium (GIRK2) channels are also implicated in analgesic action of opioid drugs (Figure 16) [158]. This hypothesis is supported by the observations that the analgesic effects of opioids were absent in GIRK2 null-mutant mice [159,160] or by OPR antagonist [161]. Alcohol exposure augments the opioid’s analgesic response by co-activating both OPR and GIRK2 channel activations [161,162]. Unlike the opioid-induced analgesia, the NMDAR-mediated analgesia may occur independently of GIRK2 channels are not modulated by alcohol exposure [162].

Kranzler et al. [163] and Zhang et al. [164] have shown that the A118G variant of the MOR1 (OPRM1) gene may be an obvious candidate mediating alcohol-induced analgesia. A118G carriers experience attenuated pain sensitivity that may alter analgesic responses to alcohol [141]. The A118G or val158met polymorphism of the catechol-O-methyl-transferase (COMT) gene could be a possible link between alcohol’s analgesia and reinforcement activities [165,166]. The val158met carrier exhibit higher COMT levels, lower DA_ergic_ neurotransmission, elevated activation of the MORs [167,168], and suppressed MOR NT response to pain [169].

**Figure 13:**
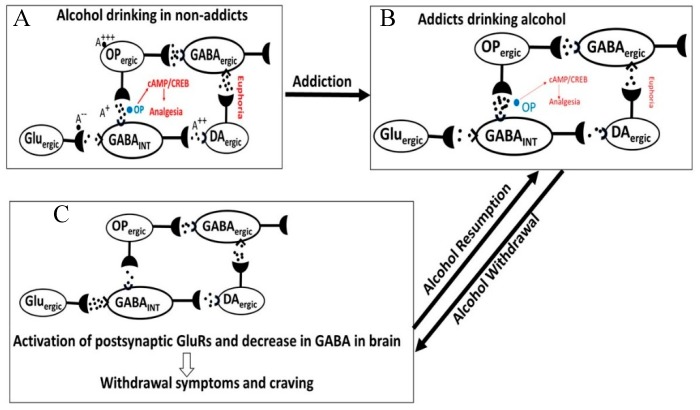
Possible mechanisms underlying the reinforcing and analgesic effects of alcohol exposure. (**A**) Acute alcohol exposure induces eOP release but inhibits Glu release from Glu_ergic_ neurons, resulting in suppression of GABA_ergic_ neurons. Downregulation of GABA_ergic_ neurons disinhibits DA_ergic_ neurons, resulting in an increase in DA release and ensuing reinforcement. Opioid exposure induces analgesia via cAMP/CREB signaling. (**B**) In addicted subjects consuming alcohol, OP_ergic_, Glu_ergic_ and GABA_ergic_ neurons respond like the non-addicted subjects, but DA_ergic_ neurons and the analgesic pathway are less responsive. Cumulatively, addicted subjects drinking alcohol exhibited poor opioid-induced analgesia and euphoria. (**C**) Alcohol abstinence in addicted subjects result in hyperactivity of Glu_ergic_ but downregulation of GABA_ergic_ neurons, causing neuronal excitation and the withdrawal symptoms. Alcohol resumption restores opioid’s analgesic potency but to a lesser degree, but eOP release is restored.

Possible CNS mechanisms underlying the addictive effects of opioids alone or in combination with alcohol are hypothesized in Figure 13. Acute alcohol exposure causes reinforcing (euphoria, red font) and weak analgesia, while acute opioid exposure (blue font) causes strong analgesia (Figure 13A red font). Acute alcohol activates DA_ergic_ neurons, thus releasing endogenous opioids (eOPs) that inhibits GABA_ergic_ activity either by directly binding to the OPRs or via inhibiting Glu release from the Glu_ergic_ neurons [170]. A decrease in GABA disinhibits postsynaptic DA_INT_ neurons resulting in an increase in DA release in NAc causing reinforcing and pleasure effects. However, acute exposure to synthetic opioids such as morphine directly activates OPR-signaling, resulting in potent activation of cAMP signaling and ensuing analgesia, with weaker reinforcing [171].

The addictive effects of alcohol and opioids are mediated by a common addiction pathway (Figure 13B) [172,173,174]. In alcoholic subjects, alcohol exposure reduces release of eOPs from OP_ergic_ neurons, but activates Glu release from Glu_ergic_ neurons, resulting in an increase in GABA_ergic_ activity and GABA release. As shown in Figure 13C, alcohol withdrawal causes further increase in Glu_ergic_ activity and decrease in GABA_ergic_ activity. This results in amplification of the withdrawal symptoms. Alcohol resumption establishes homeostasis by increasing GABA_ergic_ activity, while Glu_ergic_ activity remains elevated.

**Figure 14:**
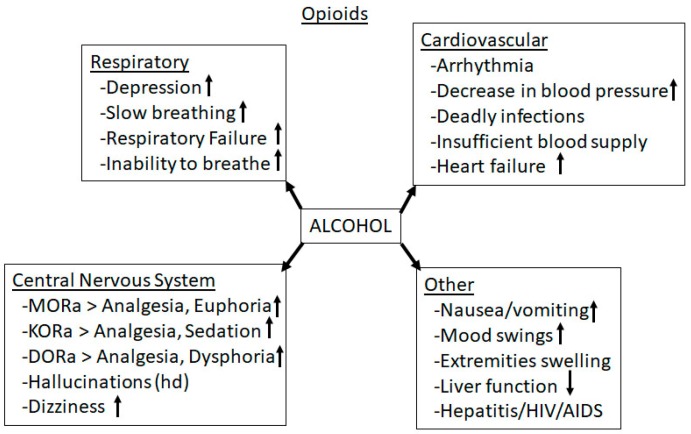
Effects of Alcohol exposure on opioid’s pharmacokinetics, cardiovascular function, CNS functions and prenatal effects.

Taken together, these observations indicate that alcohol and opioid drugs have numerous common behavioral effects, including sedation, motor depression, and rewarding experiences, possibly related to the effects of alcohol administration on release of eOP peptides [175,176]. An increase of eOP peptides increase alcohol consumption that is blocked by nonselective opioid antagonists such as naloxone and naltrexone [177,178]. Possible adverse effects of alcohol on opioids are shown in Figure 14.

### 3.5. Alcohol-Cannabis Interactions

**Figure 15:**
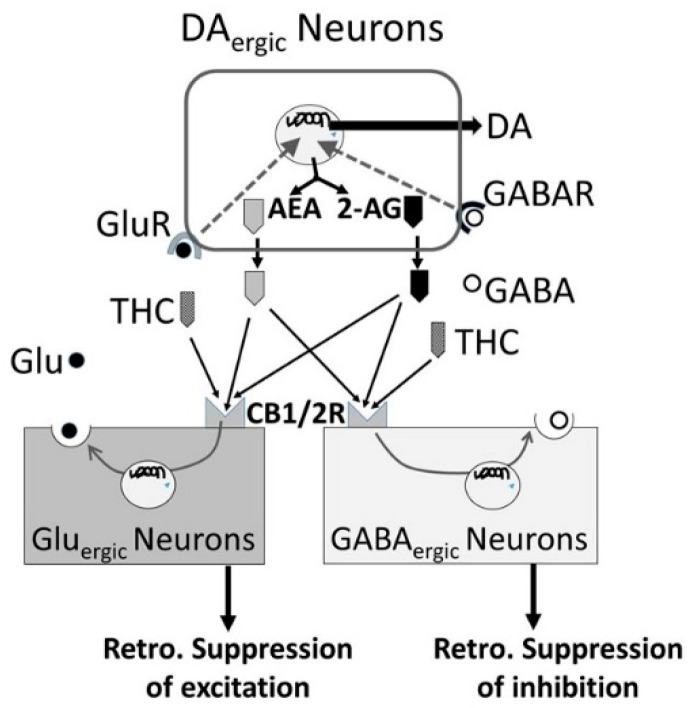
Schematic description the endocannabinoid receptor signaling. Endogenous endocannabinoids, N-arachidonoy-lethanolamine (anandamide or AEA) and 2-arachido-noylglycerol (2-AG) released by the VTA neurons, bind to CB1 and CB2 receptors on Glu_ergic_ and GABA_ergic_ neurons, resulting in release of Glu and GABA, respectively. Glu and GABA bind to their respective receptors on DA_ergic_ neurons and induce DA release. Cannabis such as THC compete with ARA and 2-AG for CB1 (AEA >> 2-AG) and CB2 (AEA ≈ 2-AG) receptors and disrupts normal endocannabinoid retrograde signaling from DA_ergic_ neurons.

Δ^9^-Tetrahydrocannabinol (THC), the main psychoactive component of cannabis [179], elicits its acute effects via the endocannabinoid (eCB) type 1 (CB_1_) receptor (CB_1_R) [180]. THC has been linked to the rewarding aspects and cognitive impairments of cannabis (Figure 15). 2-Arachidonoylglycerol (2-AG), produced by diacylglycerol lipase (DAGL) in DA_ergic_ VTA neurons [181], acts on CB_1_Rs on nearby Glu_ergic_ and GABA_ergic_ terminals. CB_1_Rs robustly inhibit GABA inputs onto VTA DA cells. CB_1_Rs are also localized on Glu_ergic_ terminals synapsing on VTA DA neurons where eCBs mediate retrograde suppression of excitation. Thus, eCBs fine-tune the activity of the mesolimbic DA projections through modulating both excitatory and inhibitory signaling [182]. THC exposure disrupts the eCB retrograde signaling system and produces complex, diverse and potentially long-term effects on the DA_ergic_ system including increase in nerve firing and DA release in response to acute THC. However, DA_ergic_ blunting may be associated with long-term use [183,184].

**Figure 16:**
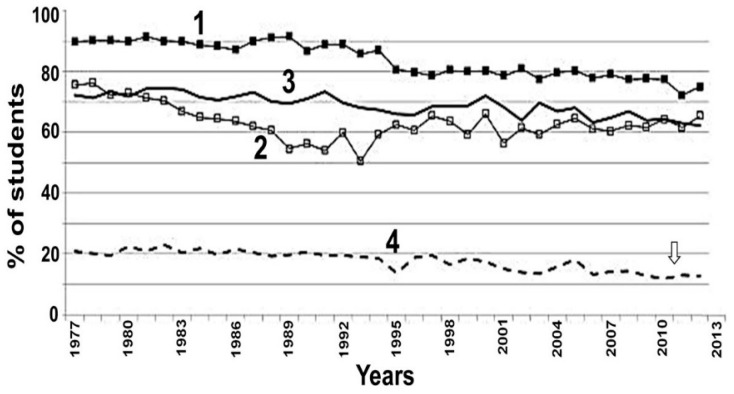
Prevalence of alcohol and cannabis use among high school students is shown from 1976–2011. Plots 1 and 2 show percentage of students using alcohol and cannabis, respectively, for last 30 days, while plots 3 shows regular cannabis use accompanied with alcohol use some time. Plot 4 shows the number of students in which cannabis use was almost always associated with alcohol use. [Data from [190] were used in this plot.

Alcohol and cannabis, being neuro-inhibitory agents, share many behavioral abnormalities such as euphoria, analgesia, sedation, hypothermia, cognitive and motor dysfunctions, etc. [185] Therefore, combination of alcohol and marijuana in occasional cannabis users may additively alter the magnitude of cognitive and motor impairments [186,187]. However, chronic cannabis use may develop tolerance to the impairing effects of cannabis and/or alcohol. Studies have shown that approximately 58% of adolescent drinkers also use cannabis [188], contributing to frequent comorbidity between alcohol and cannabis use disorders [189]. Figure 16 [190] shows 30-day trends in alcohol and cannabis use prevalence (1976–2011) among high school students. Plots 1 and 2 show percentage of students using alcohol and cannabis, respectively, for the last 30 days, while plots 3 regular cannabis uses accompanied by alcohol use some time and plot 4 shows cannabis use was almost always associated with alcohol use.

**Figure 17:**
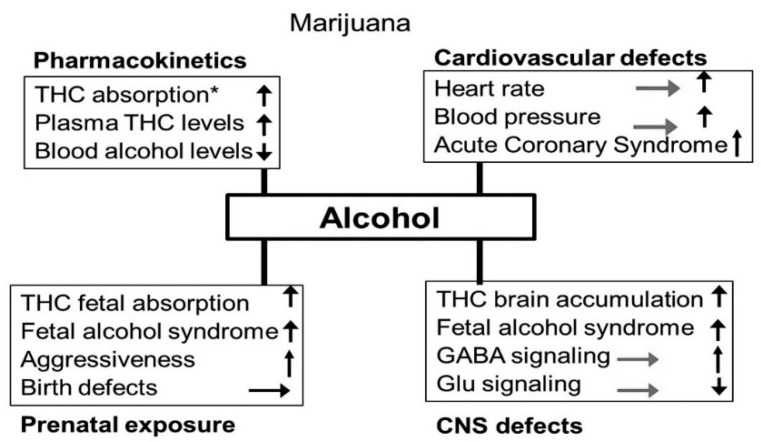
Effects of alcohol exposure on cannabis’s pharmacokinetics, cardiovascular function, CNS functions and prenatal effects.

Figure 16 suggests that a sizable proportion of US high school seniors used a combination of alcohol and cannabis in social use situations. Alcohol and cannabis use during adolescence is of concern because the introduction of drug combinations early may disrupt healthy brain development [191,192]. Studies have shown that the hippocampus (a region associated with learning and memory formation [193]) may be particularly vulnerable to structural damage caused by heavy alcohol and/or cannabis use, especially during adolescence. Aloi et al. [194] have demonstrated differential patterns of dysfunction associated with alcohol use disorder (AUD) and cannabis use disorder (CUD) symptoms. Elevated severity of AUD symptoms was associated with (i) an *increased* amygdala response to positive relative to neutral stimuli and (ii) a *decreased* responses associated with behavioral inhibition and executive attention during incongruent and congruent trials, while elevated CUD symptomatology was associated with *increased* responses in the posterior cingulate cortex, precuneus, and inferior parietal lobule for incongruent relative to congruent and view trials. This suggests that correlates of AUD symptomatology may differ from those of CUD symptomatology. Therefore, a combination of AUD and CUD may additively cause greater brain damage than AUD or CUD individually as summarized in Figure 17.

Earlier studies [195,196,197] have identified mechanistic links between the effects of alcohol and cannabinoids, both enhanced DA levels in the NAc by activating DA_ergic_ neurons in the VTA from which the mesoaccumbal DA-mediated pathway originates. Hungund et al. [198] showed that alcohol did not cause the release of DA in CB_1_^−/−^ mice or SR141716A, a selective cannabinoid receptor antagonist, administered wild-type mice. Cohen et al. [199] showed that SR141716A reduced alcohol consumption, possibly via reducing DA release in the NAc in mice. These results strongly suggest that administration of cannabis and alcohol may additively enhance DA release the NAc. Guillot et al. [200] showed that, among people using cannabis and alcohol, the interplay between social anxiety and coping-oriented motives for using one substance (such as cannabis or alcohol) may pose difficulties in refraining from other substances such as alcohol or tobacco). Therefore, it is important to tailor multi-substance treatments to specific needs when a single-substance intervention may not be effective.

**Figure 18:**
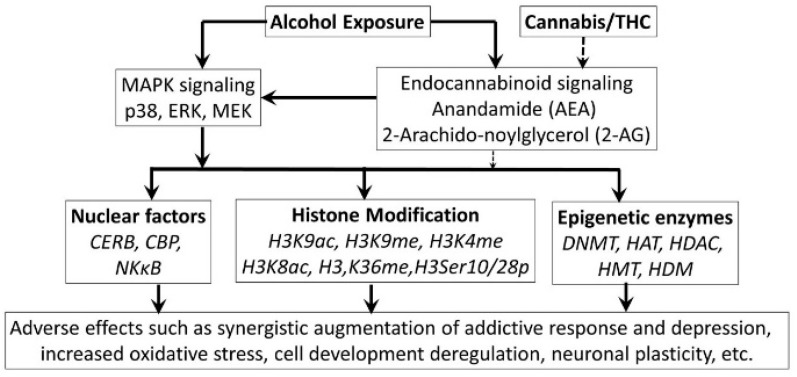
Possible epigenetic mechanisms for alcohol—cannabis interactions. Alcohol and cannabis both modulate CB1 and CB2 receptors, resulting in activation of the MAPK signaling pathways, that further activates (i) nuclear factors CREB and NF-κB, (ii) histone modifications, and (iii) DNA methylation mediated by the epigenetic enzymes. This leads to altered gene expression and cell functionality through apoptosis, oxidative stress, plasticity or immuno-modulation.

Although the molecular basis of alcohol-cannabis interaction is not yet known, recent studies have proposed epigenetic mechanisms (nuclear factors, histone modifications, and DNA methylation) to be important in determining consequences of the interaction [201,202,203]. A critical clue for alcohol-cannabis interaction was provided by the following observation: CB1 receptor knockout mice or those treated with CB1 antagonist exhibited markedly reduced voluntary alcohol consumption, possibly due to lack alcohol-induced DA release in the NAc [204]. Subbanna et al. [205] reported that post-natal alcohol exposure induced neonatal neurodegeneration possibly by enhancing CB1 exon1 activity through upregulated histone H4K8 acetylation and downregulated H3K9 methylation. Another study showed that perinatal alcohol exposure impaired DNA methylation through downregulation of DNA methyl transferases DNMT1 and DNMT3A in the neonatal brain and that such deficiencies were absent in CB1 receptor null mice [206]. Taken together, these reports suggest the potential of epigenetic overlap between alcohol and cannabis activities. Figure 18 summarizes the epigenetic mechanisms of alcohol and cannabinoid activity and possible overlaps between the two substances at the epigenetic level [207,208].

### 3.6. Alcohol-GHBA Interactions

**Figure 19:**
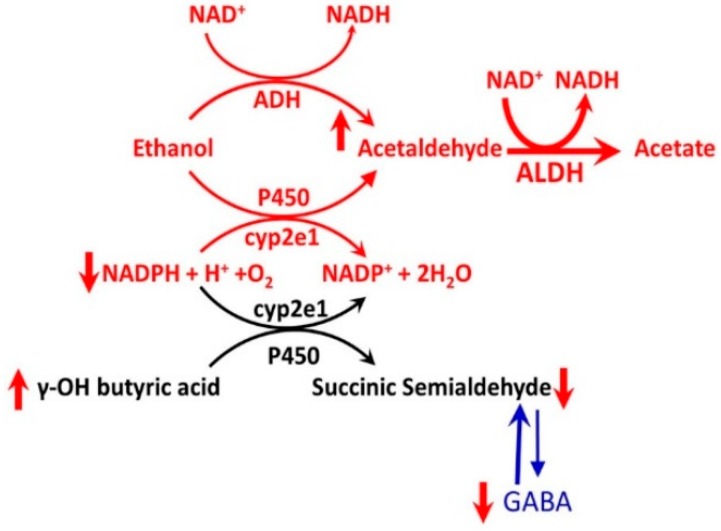
Pharmacokinetic mechanism of alcohol-GHB interaction. GHB is primarily metabolized to succinic semialdehyde (SSA) by a P450 mediated NAD(P)^+^-linked oxidation catalyzed by GHB dehydrogenase (GHBD). SSA is further metabolized to succinic acid, a citric acid cycle substrate. In case of alcohol-GHB co-exposure, alcohol competes with GHB for the enzyme’s binding sites, resulting in a decrease in GHB metabolism. However, for exogenously administered GHB, it is unclear whether co-administration with alcohol results in increased GHB or alcohol plasma concentrations.

GHB is a natural sedative with the potential to be used as a recreational drug [209,210]. The popularity of GHB as a drug of abuse has grown recently [211]. Alcohol has been shown to enhance the sedative effect of GHB in humans and animals [212,213]. Co-administration of GHB and alcohol induces sedation stronger than the sum of the sedation induced by the individual substances [214], possibly due to a pharmacokinetic interaction resulting in an increased concentration at the site of action (Figure 19).

**Figure 20:**
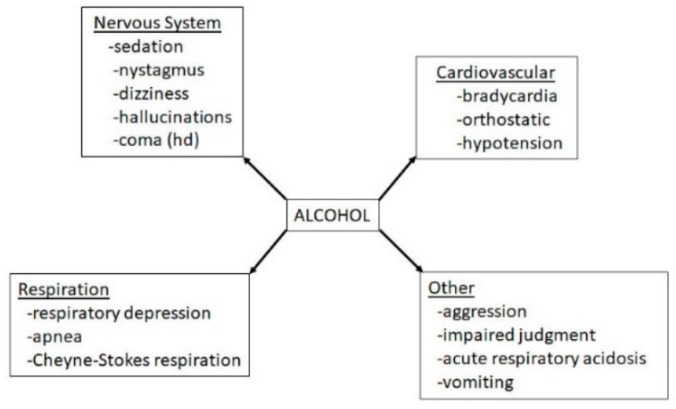
Effects of alcohol exposure on GHBA’s pharmacokinetics, cardiovascular function, CNS functions and prenatal effects.

To understand the effects of alcohol on the pharmacokinetics of GHB, it is important to understand alcohol’s interaction with the metabolic system. Studies have shown that, at lower alcohol concentrations, only about 10% of the consumed alcohol undergoes CYP-mediated first-pass metabolism in liver. Since alcohol and GBH compete for CYP2E1 (GHBD), alcohol, depending on its concentration, reduces GBH degradation and ensuing increase in its blood concentrations [215]. Chronic, heavy alcohol consumption induces the activity of CYP2E1, resulting in a decrease in GHB concentrations. The adverse effects related to GHB ingestion are shown in Figure 20 [216]. Overall adverse effects depend on the variability among users and the inherent variability in street manufacturing [217]. This makes GHB a highly dangerous drug to consume. It exhibits a steep dosage-response curve, thus, exceeding the intoxicating dose can result in severe adverse effects occurring within 15 minutes of ingestion of GHB [218,219].

## 4. Conclusions

**Figure 21:**
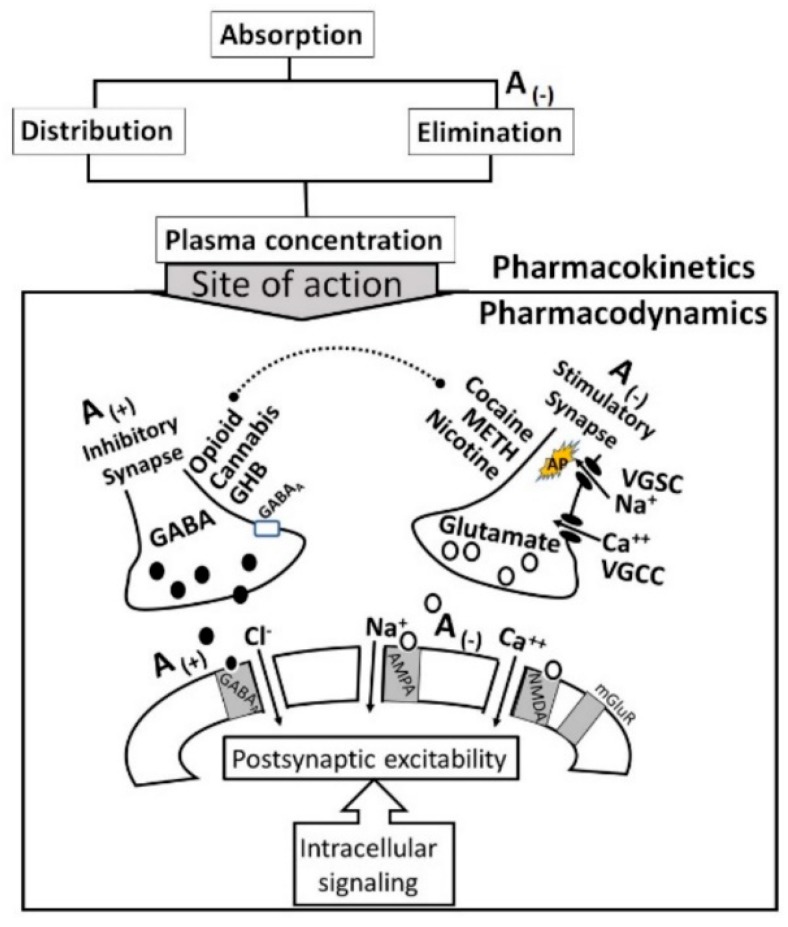
Proposed mechanisms underlying alcohol’s interaction with co-abused drugs. Alcohol (referred as alcohol in this article) is rapidly absorbed and distributed. It is metabolized to acetaldehyde by ADH and CYP enzymes, then to acetate by ALDH. In this process, alcohol competes with the co-administered drugs and suppresses their elimination, thus increasing the drug’s plasma concentration and altering its pharmacokinetics (increase in C_max_ and AUC, decrease in CL). Chronic alcohol exposure activates the liver CYP enzymes, resulting in a decrease in the drug’s plasma concentrations, with a decrease in its efficacy. Alcohol, in addition to altering pharmacokinetics of a drug, also alter its pharmacodynamics by activating GABA_ergic_ and OP_ergic_ presynaptic neurons (A_(+)_, but inhibiting Glu_ergic_ presynaptic neurons (A_(-)_). Post-synoptically, alcohol activates GABA_A_R mediated influx of Cl^−^ ions, but inhibits AMPAR-mediated Na^+^ ions and NMDAR-mediated Ca^2+^ ions, resulting in reduced excitability via IPSP. Therefore, alcohol may augment the effects of inhibitory drugs (opioids, cannabis and GHB) but suppress the effects of excitatory drugs (cocaine, METH or nicotine). Chronic alcohol exposure may have opposite effects not shown in this figure. Abbreviations: A_(+):_ positive alcohol effects, A_(−):_ negative alcohol effects, VGSE: voltage-gated sodium ion (Na^+^) channels that may be attenuated by alcohol’s IPSP, VGCC: voltage-gated calcium ion (Ca^2+^) channels that may be attenuated by alcohol’s IPSP, GABA_A_R mediated Cl^−^ channels that may be additively/synergistically activated by alcohol’s IPSP, inhibitory neurotransmitters, excitatory. neurotransmitters, 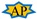 action potential.

Co-abuse of alcohol with drugs of abuse (psychostimulants (cocaine, METH and nicotine) and inhibitors (opioids, cannabis and GHBA) and medications) is a serious health problem the society faces today. People abuse multiple drugs possibly due to the perception of potentiated euphoric and pleasure effects and decreased adverse subjective effects. The negative consequences of alcohol and psychostimulant co-abuse may include a decrease in antioxidant enzymes, disruption of learning and memory processes, cerebral hypo-perfusion, neurotransmitters depletion as well as potentiated drug-seeking behavior. As summarized in Figure 21, alcohol activates inhibitory GABA_ergic_ and OP_ergic_ neurons, but inhibits excitatory Glu_ergic_ neurons. Thus, alcohol additively or synergistically augments inhibitory signaling by opioids, cannabis and GHB, but suppresses stimulatory signaling by cocaine, METH and nicotine. Alcohol may also modify the liver CYP enzymes, thus modifying the drugs plasma concentrations. Taken together, alcohol may modify both the pharmacokinetics and pharmacodynamics of co-abused drugs. Therefore, alcohol-drug interaction must be considered when developing alcoholism therapy.

## Figures and Tables

**Table 1:** Effects of alcohol on cocaine and benzoylecgonine pharmacokinetic parameters [33].

Indices	Oral Cocaine	Oral Cocaine + Alcohol	Intravenous Cocaine	Intravenous Cocaine + Alcohol
AUC_0–α_ (mg·min/L)	15.0 ± 4.7 *^×^	58.0 ± 10	83.1 ± 4.7 ^×^	110.3 ± 22.5
CL (L/min)	5.6 ± 1.8 *^×^	1.6 ± 0.35	1.0 ± 1.8 ^×^	0.74 ± 0.2
C_max_ (ng/mL)	116.0 ± 98 *^×^	331.0 ± 131	2677 ± 98	2885 ± 702
T_max_ (min)	83.6 ± 46	99.8 ± 32.5		
T_1/2_ (min)	85.2 ± 6.6	84.2 ± 9.1	75.0 ± 6.6 *^×^	84.0 ± 8.2
F	0.2 ± 0.05 ^×^	0.7 ± 0.17		
CE C_max_ (ng/mL)	ND	30.9 ± 7.3	ND	ND
BE AUC_0–α_ (mg·min/L)	172.0 ± 46 *^×^	410.0 ± 82	375.0 ± 46	407.0 ± 110
BE/cocaine AUC_0–α_	11.9 ± 3 *^×^	7.1 ± 1.5	4.9 ± 3	3.7 ± 0.6

AUC: area under the plasma concentration–time curve, CL: clearance, C_max_: maximum concentration, T_1/2_ elimination half-life, T_max_: time to C*max*, BE: benzoylecgonine. * *p* < 0.05 compared with intravenous cocaine, ^×^ *p* < 0.05 compared with corresponding alcohol group given by the same route.

**Table 2:** Pharmacodynamic parameters defining the effects of alcohol on Cocaine’s adverse effects in Wistar Rats (Mean ± SE, *n* = 8) [33].

Pharmacodynamic Parameters	Cocaine (ip) ^a^ + Normal Saline	Cocaine (ip) ^a^ + Alcohol (po)
A. Neurochemical		
E_max_ (% of baseline)	850 ± 200	1550 ± 640
EC_50_ (ng/mL)	3400 ± 580	2000 ± 650
N	1.23 ± 0.17	2.31 ± 0.29 ^b^
B. Cardiovascular		
k_in_ (% of baseline/min)	23.8 ± 5.1	36.0 ± 13.0
K_out_ (min^−1^)	0.218 ± 0.047	0.31 ± 0.11
I_max_	0.304 ± 0.033	0.307 ± 0.035
IC_50_ (mg/mL)	6700 ± 2100	5600 ± 710
R_max_ (% of baseline)	146 ± 6.9	148 ± 8.9
N	3.0 ± 1.5	3.6 ± 1.9

^a^: Cocaine dose, 30 mg/kg; alcohol dose, 5 g/kg; ^b^: Significantly different from the cocaine+normal saline treatment group (*p* < 0.05). Abbreviations: E_max_: ECF DA concentration measured at maximum change in brain ECF cocaine concentration, EC_50_: brain ECF cocaine concentration causing 50% E_max_ response, n: sigmoidicity factor, k_in_*:* apparent 0-order rate constant for response production, k_out_: 1st-order rate constant for response dissipation, I_max_: the maximum inhibition factor producing the response, IC_50_: cocaine concentration that produces 50% effect, and R_max_ maximum response.

**Table 3:** Positive (+) or negative (-) interactions among six neurotransmitter systems in the brain. Abbreviations are shown in the text [70].

NTs	Glu	GABA	5-HT	DA	NA	ACh
Glu		+	+	+	-	+
GABA	-		-	-	-	-
5-HT	+	-		+	+	-
DA	-	-	-		+	-
NA	-	-	-	+		-
ACh	+	+	+	+	+	

## Abbreviations

2-AG	2-Arachidonoylglycerol
5-HT	Serotonin
A_(+)_	alcohol’s +ve effects
A_(−)_	alcohol’s _−_ve effects
AA	arachidonic acid
AC	adenylyl cyclase
ACh	Acetylcholine
ACh_ergic_	ACh releasing neurons
ADH	alcohol dehydrogenase
ALDH	acetaldehy6de dehydrogenase
AMPA	α-amino-3-hydroxy-5-methyl-4-isoxazolepropionic acid
AUC	area under curve
AUD	alcohol use disorder
cAMP	cyclic adenosine monophosphate
CL	clearance
C_max_	maximum concentration
COMT	catechol-O-methyl-transferase
COX	Cyclooxygenase
CYP	cytochrome P450
DA	Dopamine
DA	dopamine}
DAG	Diacylglycerol
DAGL	diacylglycerol lipase
DAR1	dopamine receptor 1
DAT	dopamine transporter
DOR	delta OPRs
DPDEP	D-Pen^2^, D-Pen^5^ enkephalin, DOR agonist
EAA	excitatory amino acids
eCB	Endocannabinoid
eOP	endogenous opioids
ERK	extracellular-signal-regulated kinase
GABA	γ-aminobutyric acid
GABA_B_R	GABA B receptor
GABA_ergic_	GABA releasing neurons
GHB	γ-hydroxybutyric acid
GHBD	GHB dehydrogenase
GIRK	G protein-coupled inwardly-rectifying potassium channels
Glu	Glutamate
Glu_ergic_	glutamate releasing neurons
Gly	Glycine
IP_3_	inositol trisphosphate
KOR	kappa OPRs
LDTg	laterodorsal tegmental nucleus
LPS	Lipopolysaccharide
METH	Methamphetamine
mGluR2/3	metabolic Glu receptors 2/3
MOR	mu OPRs
NAc	nucleus accumbens
nAChR	nicotinic ACh receptor
NAPQI	*N*-acetyl-p-benzoquinone imine
NE	Noradrenaline
NMDA	*N*-methyl-d-Aspartate receptors
OP	Opioid
OPR	opioid receptors
OPRM1	A118G variant of the MOR1
PIP2	poly inositol diphosphate
pptg	pedunculopontine tegmental nucleus
SERT	serotonin transporter
sOP	synthetic opioids
SSA	succinic semialdehyde
T	elimination half-life
THC	Δ^9^-Tetrahydrocannabinol
t_max_	time to C_max_
TMU	1,3,7-trimethyluric acid
U50488H	KOR agonist
VGSC	voltage gated sodium ion (Na^+^) channel
VGCC	voltage gated calcium ion (Ca^2+^) channel
VTA	ventral tegmental area
α7 and α4β2	nicotinic receptor subtypes
